# Recovery of Olfactory Function Induces Neuroplasticity Effects in Patients with Smell Loss

**DOI:** 10.1155/2014/140419

**Published:** 2014-12-03

**Authors:** Kathrin Kollndorfer, Ksenia Kowalczyk, Elisabeth Hoche, Christian A. Mueller, Michael Pollak, Siegfried Trattnig, Veronika Schöpf

**Affiliations:** ^1^Department of Biomedical Imaging and Image-Guided Therapy, Medical University of Vienna, Waehringer Guertel 18-20, 1090 Vienna, Austria; ^2^Department of Pediatric and Adolescent Medicine, Medical University of Vienna, Waehringer Guertel 18-20, 1090 Vienna, Austria; ^3^Department of Otorhinolaryngology, Medical University of Vienna, Waehringer Guertel 18-20, 1090 Vienna, Austria; ^4^High-Field MR Center, Department of Biomedical Imaging and Image-Guided Therapy, Medical University of Vienna, Waehringer Guertel 18-20, 1090 Vienna, Austria

## Abstract

The plasticity of brain function, especially reorganization after stroke or sensory loss, has been investigated extensively. Based upon its special characteristics, the olfactory system allows the investigation of functional networks in patients with smell loss, as it holds the unique ability to be activated by the sensorimotor act of sniffing, without the presentation of an odor. In the present study, subjects with chronic peripheral smell loss and healthy controls were investigated using functional magnetic resonance imaging (fMRI) to compare functional networks in one of the major olfactory areas before and after an olfactory training program. Data analysis revealed that olfactory training induced alterations in functional connectivity networks. Thus, olfactory training is capable of inducing neural reorganization processes. Furthermore, these findings provide evidence for the underlying neural mechanisms of olfactory training.

## 1. Introduction

The neural plasticity of the human brain has been investigated extensively over the last several decades [1, 2]. Neuroplasticity can be observed not only after functional loss due to stroke, brain tumors, or sensory deprivation [3], but also after the acquisition or optimization of sensory function as a result of learning or experience [4]. The olfactory system exhibits extraordinary plasticity, due to mechanisms that have been extensively investigated at the cognitive as well as the cellular level [5, 6]. A recently published study indicated that human olfactory acuity, as well as primary cortical odor representations, persists at normal levels despite acute nasal occlusion [7]. It is assumed that these normal performance levels are maintained by compensatory top-down mechanisms. The mechanisms of neural plasticity in the olfactory system are of particular interest, as smell loss is among the first symptoms in neurodegenerative disorders, such as Alzheimer's or Parkinson's disease [8, 9]. In addition, there is a loss, or at least a reduction, in olfactory function in many neurological conditions [10]. Thus, neural plasticity with regard to olfactory loss may hold wide-spread implications for brain function far beyond olfactory perception.

Olfactory training is a promising therapeutic treatment for olfactory loss and is particularly successful in patients with smell loss after upper respiratory tract infection [11, 12]. Although the efficacy of an olfactory training program has been investigated in diverse patient groups with olfactory dysfunction [13–15], the neuronal basis of the olfactory training remains poorly understood.

The olfactory system holds the unique ability to be activated by the sensorimotor act of sniffing, which is characterized by a short and deep intake of breath through the nose, without the presentation of an odor [16, 17]. This special feature of the olfactory system permits the investigation of network alterations caused by complete peripheral sensory loss.

We, therefore, aimed to investigate modifications of functional connectivity caused by olfactory training in the left and right piriform cortices (PIR), which are among the major olfactory areas. To test this, we performed functional magnetic resonance imaging (fMRI) in anosmic patients with long-term smell loss due to infection before and after a 12-week olfactory training period. We hypothesized that training induces alterations of functional connectivity of the PIR.

## 2. Materials and Methods

### 2.1. Subjects

Eleven patients with smell loss after an upper respiratory tract infection participated in this study. Four anosmic patients had to be excluded from the data set, due to incomplete fMRI measurements, resulting in a total of seven patients with smell loss (four females, three males; mean age, 41.6 years; SD 12.9), with a mean disease duration of 4.6 years (SD 3.2) included in the analysis. Only patients diagnosed with anosmia, the complete loss of olfactory function, were included in this study [18]. All participants had no history of neurological or psychiatric diseases. The study was approved by the Ethics Committee of the Medical University of Vienna. All subjects were informed about the aim of the study and gave their written, informed consent prior to inclusion.

### 2.2. Experimental Procedure

**Figure 1:**
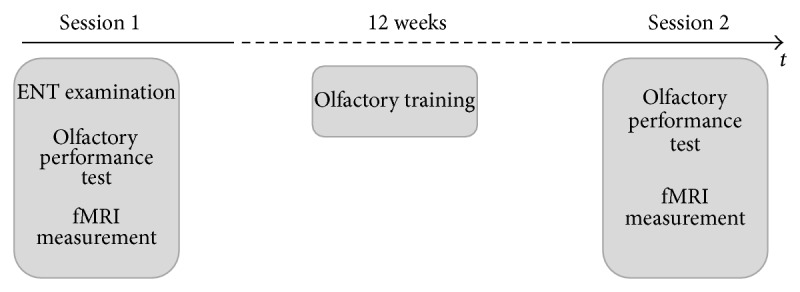
Schematic description of experimental procedure.

All patients completed two testing sessions (see Figure 1). In the first session, all patients were examined by an ENT, including an endoscopic examination of the nasal cavity, to determine the cause of olfactory dysfunction. Further, measurement of olfactory function, as described below, was performed to assess the severity of olfactory dysfunction. In the next step, fMRI measurement, using a sniffing paradigm, was performed. Following the fMRI session, all patients were instructed to perform the olfactory training over a period of 12 weeks at home. After completing the training, patients performed olfactory testing and fMRI measurement identical to the first testing session.

### 2.3. Olfactory Performance Measurement

Olfactory performance was assessed using the Sniffin' Sticks test battery (Burghart Instruments, Wedel, Germany) a clinically approved test battery, including three subtests designed to test orthonasal chemosensory function: detection threshold; odor discrimination; and odor identification. This battery uses pen-like devices for odor presentation [18–20]. The olfactory detection threshold of *n*-butanol was assessed using a single-staircase, three-alternative, forced-choice procedure. In the second step, odor discrimination ability was obtained using 16 triplets of odorants (two pens contained the same odorant; the third pen contained an odd odorant). The participants' task was to detect the odd pen in a forced-choice procedure. The odor identification task is composed of 16 common odors presented in a multiple-choice answering format, consisting of a list of four descriptors for each odor. Scores for the detection threshold range from 1 to 16, and, for the other two subtests, a score between 0 and 16 can be achieved. The results of all three subtests are summed to obtain a TDI (Threshold-Detection-Identification) score. We defined anosmia based upon clinical definitions [18]. Specifically, anosmia was defined by a TDI score of 17 or less.

### 2.4. Sniffing Paradigm

The paradigm for the fMRI experiment consisted of five sniffing blocks and five normal breathing blocks, with each block consisting of eight cycles and duration of 32 seconds (Figure 1). Each sniff of the sniffing block was characterized by a short and deep intake of nonodorized air through the nose. Before each scanning session, subjects were trained to perform this paradigm correctly. No odor was presented during the session. For temporal standardization, the subject's breathing cycles were guided by auditory stimuli.

### 2.5. Olfactory Training

Olfactory training was performed over a period of 12 weeks [11]. Following the initial fMRI measurement, all participants had to choose four of six odors to perform the olfactory training: cinnamon (cinnamaldehyde; 30% v/v dissolved in 1,2-propanediol); vanilla (vanillin; 1 g dissolved in 1 mL 1,2-propanediol); orange (orange oil); rose (phenylethyl alcohol; PEA); menthol; and banana (isoamyl acetate; 1% v/v dissolved in 1,2-propanediol). Patients received four brown glass jars, labeled with the name of the odor (50 mL total volume) and filled with 1 mL of the respective odorant (soaked in cotton pads to prevent spilling). All patients were instructed to expose themselves twice a day to each of the four odors and take one deep sniff of every odor. Further, patients were advised to keep a diary over the training period to monitor whether the training was performed steadily. In addition, all patients were contacted weekly by an experimenter to maintain compliance and motivation over the full training period.

### 2.6. Statistics

Statistical analysis was performed using the Statistical Package for the Social Sciences (SPSS, Chicago, Illinois, USA), version 20.0. For all test scores, mean and standard deviation (SD) were calculated. To compare olfactory performance scores before and after the smell training, the nonparametric Wilcoxon test was performed due to the small sample size. The alpha level for all statistical tests was set to *α* = 0.05.

### 2.7. Imaging Methods

fMRI measurements were performed on a 3 Tesla Trio System (Siemens Medical Solution, Erlangen, Germany) using a 32-channel head coil. Functional images were acquired using an optimized 2D single-shot, gradient-recalled, echo-planar imaging (EPI) sequence, including online distortion correction with point-spread function-mapping [21]. Thirty-six slices (2.7 mm thickness, 0.5 mm gap), aligned parallel to the AC-PC line, were acquired, with an echo time (TE)/repetition time (TR) of 32/2000 ms and a field of view (FOV) of 210 × 210 mm.

### 2.8. fMRI Data Analysis

fMRI data were preprocessed using SPM12b (http://www.fil.ion.ucl.ac.uk/spm/), implemented in MATLAB (Matlab 7.14.0, Release 2012a, Mathworks Inc., Sherborn, MA, USA), which included motion correction, spatial normalization to an MNI template, and spatial smoothing. Seed regions for functional connectivity analysis, that is, the left and right piriform cortices, were selected on a standard brain using the WFU PickAtlas [5, 6]. Those regions are primarily responsible for olfaction and are known to be involved in sniffing processing as well [6, 17, 22]. ROI-to-ROI functional connectivity analysis was performed using the CONN toolbox [23] (http://www.nitrc.org/projects/conn) implemented in MATLAB. Using CONN, additional preprocessing steps were performed. First, nuisance parameters that were extracted from the motion correction process were regressed out. Further, the mean time course from the preselected seed region was correlated with the time course of all other ROIs in the brain (Brodmann areas). The results provided separate spatial maps of functional connectivity within the preselected seed region for both measurement time points.

## 3. Results

### 3.1. Olfactory Performance

All patients performed the smell training for 12 weeks. The mean time period between the two testing sessions was 13 weeks. According to their diaries, all patients performed the olfactory training regularly, twice per day. Comparison of olfactory performance measurements revealed a significant improvement in the odor detection threshold (*P* = 0.028). No significant difference between the two testing sessions was obtained for the odor discrimination task (*P* = 0.916) or the odor identification task (*P* = 0.673). Detailed results of the olfactory performance testing are presented in Table 1. In six out of seven patients an improved odor threshold performance was determined in the second testing session.

### 3.2. fMRI Results

**Figure 2:**
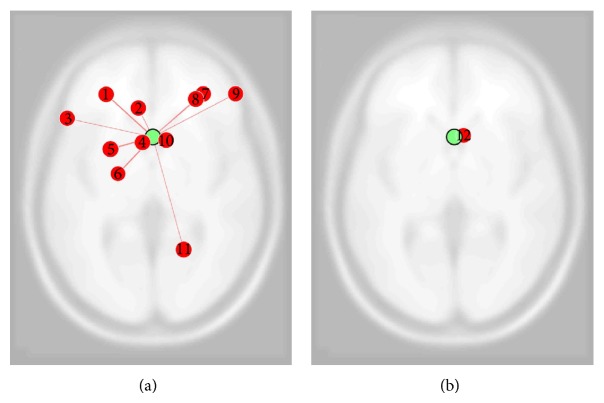
Functional connectivity during sniffing for anosmic patients before (a) and after (b) the smell training performed over 12 weeks, overlaid on an axial template in MNI space (*P* = 0.01, uncorrected). The green dot represents the selected ROI (piriform cortex); the red dots capture the statistically significant functionally connected brain areas. (1) Dorsolateral prefrontal cortex (l). (2) Dorsal anterior cortex (l). (3) Inferior frontal gyrus (l). (4) Ventral anterior cortex (l). (5) Premotor cortex (l). (6) Posterior entorhinal cortex (l). (7) Dorsolateral prefrontal cortex (r). (8) Dorsal frontal cortex (r). (9) Dorsolateral prefrontal cortex (r). (10) Ventral anterior cortex (r). (11) Somatosensory association cortex (r). (12) Subgenual cortex (r).

Functional connectivity analysis revealed different functional connectivity of the PIR before and after the olfactory training (Figure 2 and Table 2). Prior to the olfactory training, a widespread network encompassing largely nonolfactory regions was observed. Significant connectivity from the PIR to the left and right prefrontal areas, the left inferior frontal gyrus, and the left premotor cortex was noted (Figure 2(a)). Thus, our results revealed a functionally connected network far beyond the olfactory areas. In contrast, after the olfactory training, these nonolfactory network connections dispersed; only one significant connection with the PIR was retained (Figure 2(b)).

## 4. Discussion

The results of this study revealed different functional connectivity networks for the PIR in anosmic patients before and after a 12-week olfactory training program. Before the olfactory training, a diverse network involving mostly nonolfactory regions was observed, including the prefrontal areas as well as the left inferior frontal gyrus and the left premotor cortex. After the training, these nonolfactory functional connections declined. Thus, we were able to show a neural reorganization process induced by an olfactory training program.

The high plasticity of sensory systems has been demonstrated frequently over the last several decades [1, 2]. In addition to plasticity as it relates to functional loss, such as alterations of brain structures and functional connectivity after stroke or brain tumors, the reorganization and the establishment of new connections have also been demonstrated at the cognitive [24] as well as the cellular level [25]. The mechanisms underlying the extraordinary plasticity of the olfactory system are still under investigation [5].

Training of a specific function may not only cause changes at the cognitive or behavioral level, but also induce alterations in structural [26] and functional connectivity [27] in the central nervous system. Previous studies have demonstrated that olfactory training can at least partially restore olfactory function in anosmic patients, especially in patients with smell loss after an upper respiratory tract infection [11, 12], but also in patients with Parkinson's disease [14]. Based on the results of our study, we assume that high functional plasticity of the olfactory system, especially in the PIR, is one of the major fundamentals of the success of olfactory training. As hypothesized, the recovery of olfactory function, which is reflected mainly as an improvement in the odor detection threshold, was accompanied by a transformation of the aberrant functional connectivity in the PIR that was observed in anosmic patients prior to olfactory training.

The PIR is a brain structure that is highly involved in olfactory perception, as it receives dense input from olfactory bulb projection neurons. Further projections of the PIR involve higher olfactory areas, such as the orbitofrontal gyrus, the entorhinal cortex, and the limbic system [28]. The PIR was selected as an ROI for the investigations in this study, as it is the region primarily responsible for olfaction. Furthermore, previous research has revealed that neural activation within the PIR is not solely reliant on odor stimulation. Indeed, the PIR shows neural activation induced by the sensorimotor act of sniffing alone—without the presentation of an odor [22, 29]. In addition, single-cell recordings in the PIR of mice have been detected to show action potential that is highly correlated to inhalation [30].

Sensory loss often entails functional and structural modifications of the central nervous system [31, 32]. A recently published study reported that grey and white matter volume decreases in anosmic patients compared to healthy controls [33, 34]. Furthermore, there was also a significant increase in atrophy with longer disease duration. Volume loss was detected in primarily olfactory areas, such as the PIR, but also in brain areas with more generalized function, such as the anterior cingulate cortex or the anterior insular cortex. A decrease in grey and white matter volume was detected not only in anosmic patients, but also in patients with reduced olfactory function (hyposmia) [35]. Thus, grey and white matter volume apparently is decreased by reduced sensory input. In our study, we did not focus on structural alterations induced by sensory loss, but we focused on the functional connectivity of basic olfactory structures, such as the PIR. Based on our previous structural investigations, our results indicate that there are effects of smell loss far beyond those in the olfactory processing brain areas. Understanding the extent and the mechanisms of plasticity in the olfactory system will enable insight into brain mechanisms for recovery and reorganizational processes and is particularly relevant as the loss of olfactory function is among the first symptoms in neurodegenerative disorders [10]. Based upon the results of our study, it seems that olfactory training may induce extensive reorganizational processes in more than just the olfactory areas, and thus olfactory training may strengthen higher cognitive function far beyond olfactory perception.

In addition to the central effects of olfactory training manifesting in network changes, there was a statistically significant improvement in the odor detection threshold. In contrast, in the two other smell subtests, odor discrimination and odor identification, no differences between the two measurement time points, before and after training, were obtained. Numerous previous studies have reported deficits in odor discrimination and identification abilities, whereas odor detection thresholds remained intact [36, 37]. Hedner et al. [38] investigated cognitive factors and their relation to odor identification, odor discrimination, and odor detection threshold and found that cognitive factors had a higher impact on discrimination and identification abilities. Thus, it is assumed that the odor detection threshold represents basic olfactory function, whereas odor discrimination and odor identification delineate higher olfactory functions. The results of our study indicate that olfactory training improves the most basic olfactory function and could conceivably affect higher olfactory function, such as discrimination and identification abilities, after longer training periods. However, previous studies [11] revealed an increase also in odor discrimination and odor identification, whereas no improvement was determined in our study. This finding may be caused by dissimilar study samples as previous studies included patients with olfactory dysfunction, ranging from mild hyposmia to complete anosmia, whereas in our study only patients with functional anosmia were included. The lack of improvement in higher olfactory functions may therefore conceivably be caused by the more severe olfactory dysfunction. It will be interesting for future studies to investigate the long-term effects of olfactory training, which may shed light on the impact of olfactory training on higher olfactory function. Higher olfactory function may contingently require a longer training period to recover, as the recovery of basic sensory perception is required for the regeneration of higher function. Furthermore, the investigation of effects of the olfactory training in anosmic compared to hyposmic patients should be part of future research.

In this study, we were able to show that functional connectivity underlies reorganizational processes induced by training. After a 12-week training period, all nonolfactory connections disappeared. However, during this time, no statistically significant connections to olfactory-related areas were established. At the behavioral level, statistically significant improvement was observed only at the detection threshold test. Thus, functional connectivity of the PIR with other olfactory areas may require a longer training period and the recovery of higher olfactory function. Future studies may shed light on functional connectivity alterations after longer training intervals of at least 18 weeks, as proposed by Damm et al. [12]. Training-induced behavioral improvement in olfactory performance has previously been shown not only in functional anosmic patients, but also in patients with reduced olfactory function (hyposmia), as well as subjects with normal olfactory function. Although we were able to show alterations in functional networks in patients with anosmia, potential modifications of functional connectivity patterns in patients with hyposmia should be investigated in future studies.

A limitation of this study is the small sample size of seven anosmic patients who completed all measurements. However, we performed a very strict screening procedure that included only anosmic patients with smell loss after an upper respiratory tract infection to avoid influence of different causes of smell loss. We chose to investigate postinfectious anosmic patients, as previous studies have reported that olfactory training was most successful in these patients. At the time point the study was designed the recommendation training period regarding the smell training was 12 weeks [11]; however, a multicenter follow-up study [12] revealed superior improvement of olfactory performance after a training period of 32 weeks. Therefore, future study investigating effects on other olfactory performance measures and neural patterns after longer olfactory training periods should follow.

## 5. Conclusion

The results of our study revealed training-induced modifications in functional connectivity of major olfactory areas (PIR). At the behavioral level, olfactory training mainly affected the odor detection threshold, the most basic function of olfactory performance. We included only patients with postinfectious smell loss in this study, as previous literature suggests olfactory training is most successful in this patient group [12]. Investigations of the training-induced mechanisms in olfactory-related areas in patients with olfactory loss after traumatic brain injury may provide information about dual neuroplasticity effects caused by internal (brain damage) and external (behavioral training) features.

## Figures and Tables

**Table 1:** Results of olfactory performance measurements before and after olfactory training.

	Before training mean (SD)	After training mean (SD)	*P* value
TDI score	11.82 (1.66)	13.79 (4.21)	0.128
Threshold	1.39 (0.61)	3.07 (1.98)	0.028
Discrimination	5.57 (1.27)	5.71 (1.98)	0.916
Identification	4.86 (2.04)	5.00 (2.16)	0.673

**Table 2:** Intensity of functional connectivity with the PIR.

	Anatomic label	*T*(6)	*P* value^a^
Before training	Left premotor cortex	5.57	0.001
	Left posterior entorhinal cortex	4.94	0.003
	Left dorsolateral prefrontal cortex	4.72	0.003
	Right ventral anterior cingulate cortex	4.46	0.004
	Left ventral anterior cingulate cortex	4.46	0.004
	Left inferior frontal gyrus (pars triangularis)	4.32	0.005
	Right dorsolateral prefrontal cortex	4.19	0.006
	Right dorsal frontal cortex	3.97	0.007
	Right somatosensory association cortex	3.94	0.008
	Left dorsal anterior cortex	3.72	0.009

After training	Right subgenual cortex	6.69	0.0005

^a^Uncorrected *P* value, thresholded at 0.01.
